# Comparison of the toxicities, activities and chemical profiles of raw and processed Xanthii Fructus

**DOI:** 10.1186/s12906-016-0994-3

**Published:** 2016-01-22

**Authors:** Tao Su, Brian Chi-Yan Cheng, Xiu-Qiong Fu, Ting Li, Hui Guo, Hui-Hui Cao, Hiu-Yee Kwan, Anfernee Kai-Wing Tse, Hua Yu, Hui Cao, Zhi-Ling Yu

**Affiliations:** 1 School of Chinese Medicine, Hong Kong Baptist University, Kowloon Tong, Hong Kong China; 2 National Engineering Research Center for Modernization of Traditional Chinese Medicine, Zhuhai, China; 3 Institute of Integrated Bioinfomedicine & Translational Science, HKBU Shenzhen Research Institute and Continuing Education, Shenzhen, China

**Keywords:** Xanthii Fructus, Stir-baking, Cytotoxicity, Anti-inflammation, UPLC/Q-TOF-MS

## Abstract

**Background:**

Although toxic, the Chinese medicinal herb Xanthii Fructus (XF) is commonly used to treat traditional Chinese medicine (TCM) symptoms that resemble cold, sinusitis and arthritis. According to TCM theory, stir-baking (a processing method) can reduce the toxicity and enhance the efficacy of XF.

**Methods:**

Cytotoxicities of raw XF and processed XF (stir-baked XF, SBXF) were determined by the MTT (3-(4,5-dimethylthiazol-2-yl)-2,5-diphenyltetrazolium bromide) assay in normal liver derived MIHA cells. Nitric oxide (NO) production and inducible nitric oxide synthase (iNOS) mRNA expression were measured by the Griess reagent and quantitative real-time PCR, respectively. The chemical profiles of XF and SBXF were compared using an established ultra-performance liquid chromatography/quadrupole-time-of-flight mass spectrometry (UPLC/Q-TOF-MS) method.

**Results:**

SBXF was less toxic than XF in MIHA cells. Both XF and SBXF had anti-inflammatory effects as demonstrated by their abilities to reduce nitric oxide production as well as inducible nitric oxide synthase mRNA expression in lipopolysaccharide-stimulated RAW 264.7 macrophages. Interestingly, the anti-inflammatory effects of SBXF were more potent than that of XF. By comparing the chemical profiles, we found that seven peaks were lower, while nine other peaks were higher in SBXF than in XF. Eleven compounds including carboxyatractyloside, atractyloside and chlorogenic acid corresponding to eleven individual changed peaks were tentatively identified by matching with empirical molecular formulae and mass fragments, as well as literature data.

**Conclusion:**

Our study showed that stir-baking significantly reduced the cytotoxicity and enhanced the anti-inflammatory effects of XF; moreover, with a developed ultra-performance liquid chromatography/quadrupole-time-of-flight mass spectrometry method we differentiated XF and SBXF by their chemical profiles. Further studies are warranted to establish the relationship between the alteration of chemical profiles and the changes of medicinal properties caused by stir-baking.

## Background

Xanthii Fructus (XF) is the ripe fruits of *Xanthium sibiricum* Patr. (Compositae). In *Sheng Nong’s herbal classic* (a book published 2,000 years ago), XF was first documented to be able to smooth nasal orifices (通鼻窍) and eliminate wind-dampness (祛风湿), and to be toxic. It is commonly used in managing traditional Chinese medicine (TCM) symptoms that would today be diagnosed as cold, sinusitis and arthritis [1]. To reduce the toxicity and enhance the efficacy, XF is usually processed by stir-baking (炒). In the Chinese pharmacopoeia, more than ten Chinese proprietary drugs contain XF or stir-baked XF (SBXF). Chemical analyses revealed that XF contains water-soluble glycosides, sesquiterpene lactones and phenolic acids [2]. Pharmacological studies showed that XF has various bioactivities including anti-oxidant [3], anti-bacterial [4] and anti-inflammatory [5] properties. In a carrageenan-induced hind paw edema model in rats, XF was shown to have anti-inflammatory effect as demonstrated by reducing the levels of inducible nitric oxide synthase (iNOS) and cyclooxygenase-2 (COX-2) expressions [5]. In an acetic acid-induced writhing model, SBXF was shown to have better analgesic effect than XF in mice [6]. Toxicological studies demonstrated that XF induced obvious liver damage in a long-term toxicity study in rats [7], the water extract was more toxic than the ethanol extract of XF in mice [8, 9], and the water extract of SBXF was less toxic than XF in an acute toxicity study in mice [6]. However, the comparison of the hepatotoxicities of XF and SBXF has not been conducted. Proteins and water-soluble glycosides have been thought to be the main toxic substances of this herb. Denaturing the toxic proteins by stir-baking has been proposed as one of the reasons for reducing its toxicity [10]. The water-soluble glycosides carboxyatractyloside (CATR) and atractyloside (ATR) have been recognized as other two toxic components of this herb [11, 12].

To validate the impact of stir-baking on the toxicity and efficacy, in this study, we compared the cytotoxicities of XF and SBXF in non-tumorigenic and immortalized human liver cells (MIHA), and their anti-inflammatory effects in lipopolysaccharide (LPS)-stimulated Raw 264.7 macrophages. In an attempt to uncover the chemical basis behind the potential changes of medicinal properties caused by stir-baking, we compared the chemical profiles of the water extracts of XF and SBXF using an established UPLC/Q-TOF-MS method.

## Methods

### Chemicals and regents

Carboxyatractyloside potassium salt (C_31_H_43_O_18_S_2_K_3_) was purchased from Merck (Merck Millipore, Taiwan). Atractyloside potassium salt (C_30_H_44_O_16_S_2_K_2_), chlorogenic acid (C_16_H_18_O_9_ ), caffeic acid (C_9_H_8_O_4_), 1,5-O-Dicaffeoylquinic acid (C_25_H_24_O_12_), 3-(4,5-dimethylthiazol-2-yl)-2,5-diphenyltetrazolium bromide (MTT), bacterial lipopolysaccharide (LPS) and Griess reagent were purchased from Sigma-Aldrich (Sigma, USA). All solvents for chemical analyses were purchased from RCI Labscan Ltd. (Thailand). All materials for cell culture were obtained from Life Technologies Inc. (GIBICO, USA).

### Preparation of XF and SBXF extracts

Ten batches of XF were collected from different places in China (01: Yulin, Shanxi province; 02: Shunyi, Beijing; 03: Houma, Shanxi province; 04–05: Baoding, Hebei province; 06: Handan, Hebei province; 07–10: Hong Kong), and their authentication were confirmed by the corresponding author. Voucher specimens were deposited at the School of Chinese Medicine, Hong Kong Baptist University.

SBXF preparation: Raw XF was stir-baked in a pre-heated wok. The processed herb is SBXF.

XF and SBXF extracts preparation: XF or SBXF (100 g) was reflux-extracted twice with water (1:10, w/v) for 2 h each. The combined extracts were filtered and then concentrated under reduced pressure to remove the water. The powdered XF (yield: 12.00 %) and SBXF (yield: 17.22 %) extracts were obtained by lyophilizing of the concentrated samples with a Virtis Freeze Dryer (The Virtis Company, New York, USA).

XF and SBXF fractions preparation: XF or SBXF (100 g) was reflux-extracted twice with 80 % ethanol (1:10, w/v) for 2 h each. The combined extracts were filtered and evaporated under vacuum, then suspended in water and partitioned successively with petroleum (PE), ethyl acetate (EA) and n-butanol (n-Bu). Each fractions of XF and SBXF were evaporated in a Virtis Freeze Dryer to yield the residues of PE (8.56 %), EA (5.99 %), n-Bu (16.7 %) and aqueous (68.75 %) for XF, and PE (10.18 %), EA (7.28 %), n-Bu (17.00 %) and aqueous (65.54 %) for SBXF, respectively.

### Cell culture

The non-tumorigenic and immortalized human liver cells (MIHA) and the murine macrophage cells (Raw 264.7) were obtained from the American Type Culture Collection (ATCC, Manassa, VA, USA). All cells were cultured in dulbecco’s modified eagle medium (DMEM) supplemented with 10 % heat inactivated fetal bovine serum and 1 % penicillin/streptomycin at 37 °C in humidified 5 % CO_2_ atmosphere.

### Cytotoxicity assay

MIHA cells were seeded on a 96-well plate (5000 cells/well) and allowed to adhere overnight. The cells were treated with various concentrations of the water extracts and fractions of XF and SBXF as indicated for 48 h, 20 μl of MTT solution (5 mg/ml) was added to each well and incubated for an additional 3 h. The formazan crystal formed was dissolved with 100 μl of dimethylsulfoxide (DMSO), absorbance was detected at 570 nm by a microplate spectrophotometer (BD Biosciences, USA). Results were expressed as percentages of the respective controls [13].

### Nitric oxide (NO) production assay

RAW 264.7 cells were seeded on a 24-well plate (1 × 10^5^ cells/ well) and allowed to adhere overnight. After pretreated with LPS (1 μg/ml) for 2 h, the cells were treated with different subtoxic concentrations of XF or SBXF water extract (100, 200, 300 μg/ml, cell survival >90 %) in the presence of LPS for another 24 h. NO production was determined by assaying the accumulation of nitrite, the primary stable breakdown product of NO in the culture medium, with the Griess reagent [14]. The absorbance at 540 nm was measured using a microplate spectrophotometer (BD, Bioscience USA).

### Real-time polymerase chain reaction

RAW 264.7 cells were seeded as described in Section “*Nitric oxide (NO) production assay*”. After pretreated with LPS (1 μg/ml) for 2 h, the cells were treated with different subtoxic concentrations of XF or SBXF water extract (100, 200, 300 μg/ml, cell survival >90 %) in the presence of LPS for another 18 h. Total RNA was isolated using Trizol reagent (Invitrogen, USA) according to manufacturer’s protocol [15]. Five micrograms of RNA was used for reverse transcription by oligo-dT using the SuperScript II Reverse Transcription Kit (Invitrogen, USA). The primers were designed as follows: iNOS (Sense 5’-AACGGAGAACGTTGGATTTG-3’ and anti-sense 5’-CAGCACAAGGGGTTTTCTTC-3’). To normalize the amounts of RNA in samples, a PCR reaction was also performed with primers of GAPDH (Sense 5’-AACTTTGGCATTGTGGAAGG-3’ and anti-sense 5’-TGTGAGGGAGATGCTC AGTG-3’). Real-time PCR was performed using SYBR green reaction mixture in the ABI 7500 Fast Real-time PCR System (Applied Biosystems, USA).

### UPLC/Q-TOF-MS analysis

Liquid chromatography was performed on an Agilent 1200 system coupled with an ACQUITY UPLC HSS T3 column (2.1 mm × 100 mm, 1.8 μm) maintained at 35 °C. Elution was performed with a mobile phase of A (0.1 % formic acid in acetonitrile) and B (0.1 % formic acid in water). A gradient elution of 12 % A at 0–2 min, 12–25 % A at 2–5 min, 25–40 % A at 5–7.5 min, 40–65 % A at 7.5−10 min, 65–80 % A at 10–13 min and 80−12 % A at 13–17 min was employed. The flow rate was set at 0.35 ml/min. The injection volume was 5 μl.

Mass spectrometric detection was carried out on an Agilent 6540 Q-TOF mass spectrometer (Hewlett Packard, Agilent, USA) with electrospray ionization (ESI) interface. The negative ion mode was used with the mass range set at m/z 100–1700. The conditions of ESI source were as follows: gas temperature, 300°C; drying gas (N_2_) flow rate, 8 L/min; nebulizer, 45 psi; sheath gas temperature, 350 °C; sheath gas flow, 10 L/min; capillary voltage, 4000 V; fragmentor, 140 V; skimmer voltage, 65 V; OctopoleRFPeak, 750 V. Data were collected with the LC-MS-QTOF MassHunter Data Acquisition Software Ver. A.01.00 (Agilent Technologies) and analyzed with the Agilent MassHunter Qualitative Analysis Software B.06.00, respectively.

### Statistical analysis

Each experiment was performed in triplicate and was repeated for at least three times. All results were presented as mean ± SD. The significance of differences among groups was determined by the one-way analysis of variance (ANOVA). Variance between two groups was evaluated by Student’s *t*-test. All analyses were performed using SPSS 17.0 (IBM, USA) with *p* < 0.05 as the significance level.

## Results and discussion

### Stir-baking reduced the cytotoxicity of XF in MIHA cells

**Fig. 1:**
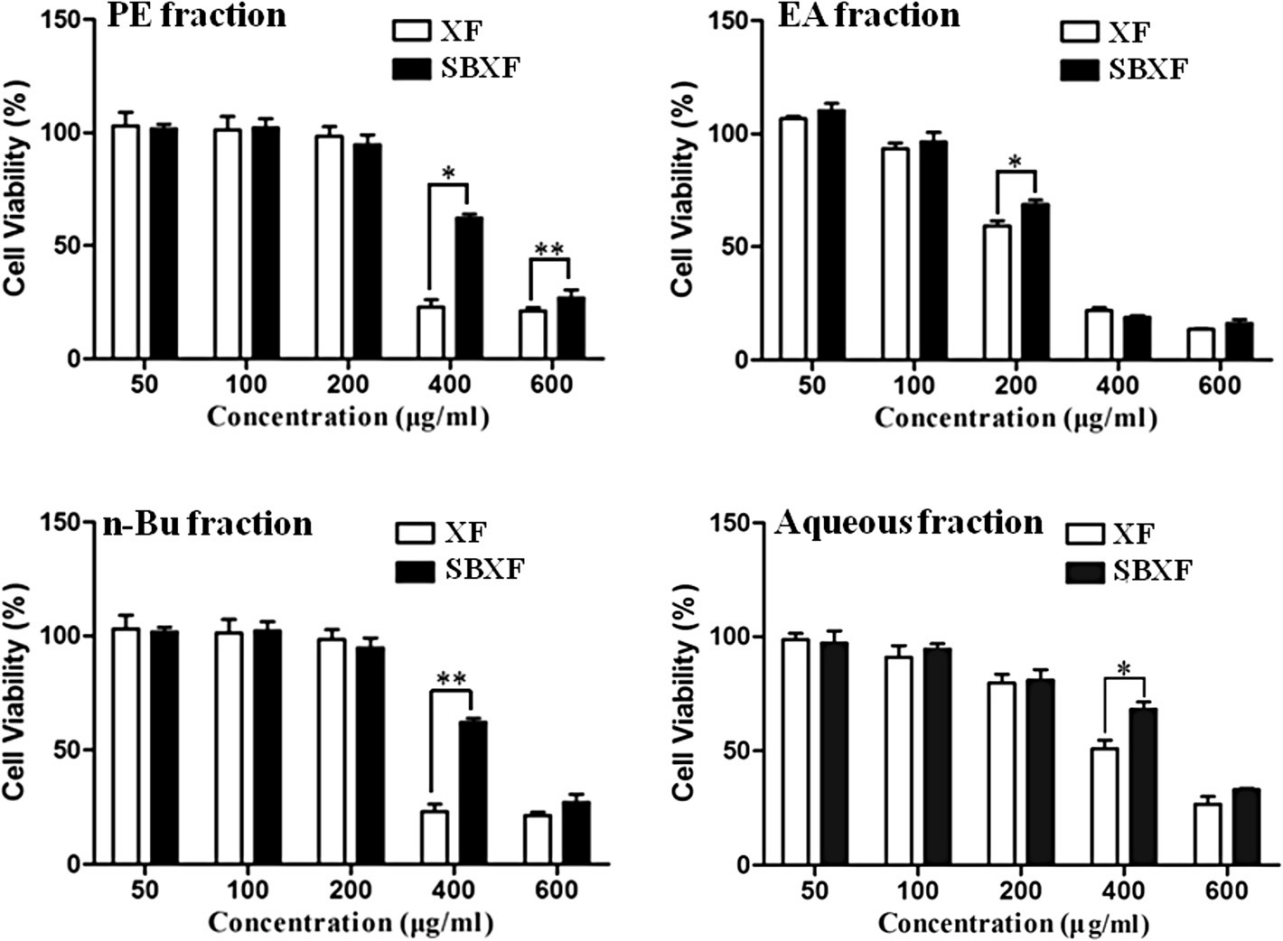
Cytotoxicities of XF and SBXF fractions in cultured MIHA cells. MIHA cells were treated with various concentrations of XF and SBXF fractions as indicated for 48 h, cell viability was determined by the MTT assay. All data were presented as mean ± SD. PE: petroleum; EA: ethyl acetate; n-Bu: n-butanol. *p<0.05, **p<0.01

It has been reported that XF has liver toxicity in animals [7], and two water-soluble glycosides have been identified to be the toxic substances [11, 12]. To compare the cytotoxicities of XF and SBXF water extracts, we used the MIHA cell model. Results showed that both XF and SBXF water extracts have no obvious cytotoxicities (data not shown). However, whole power of this herb was also used in the clinic, in order to know the cytotoxicities of the components with different polarities, we prepared fractions of an ethanolic extract of XF or SBXF and individually tested their cytotoxicities. As shown in Fig. 1, EA fractions (XF IC_50_: 231.1 μg/ml; SBXF IC_50_: 282.2 μg/ml) were more toxic than other three fractions (PE fraction: XF IC_50_: 391.8 μg/ml; SBXF IC_50_: 499.5 μg/ml; n-Bu fraction: XF IC_50_: 271.8 μg/ml; SBXF IC_50_: 512.9 μg/ml; aqueous fraction: XF IC_50_: 539.9 μg/ml; SBXF IC_50_: 560.8 μg/ml). Nevertheless, SBXF was less toxic than XF in all four fractions, suggesting that stir-baking reduced the cytotoxicity of XF in MIHA cells.

### Stir-baking enhanced the anti-inflammatory effects of XF in LPS-stimulated macrophages

**Fig. 2:**
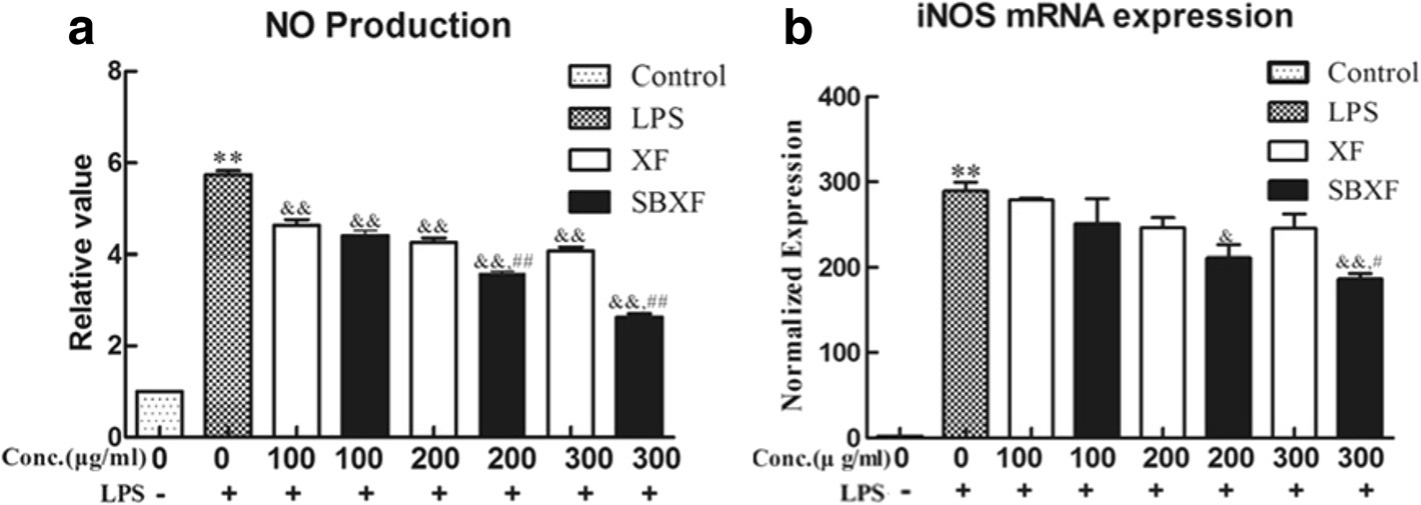
Effects of XF and SBXF on NO production (**a**), iNOS mRNA expression levels (**b**) in LPS-stimulated RAW 264.7 cells. (**a**) Raw 264.7 cells were pretreated with LPS for 2 h, then cells were treated with XF or SBXF water extract in the presence of LPS for another 24 h. NO production was determined by the Griess reagent. (**b**) Raw 264.7 cells were pretreated with LPS for 2 h, then cells were treated with XF or SBXF water extract in the presence of LPS for another 18 h. iNOS mRNA expression was assessed by real-time PCR. All data were presented as mean ± SD. **p<0.01 *vs.* control; &&*p*<0.01 *vs.* LPS; ^#^ *p*<0.05, ^##^ *p*<0.01 *vs.* XF

XF is commonly used to treat TCM symptoms that resemble cold, sinusitis and arthritis. It has a good anti-inflammatory activity. In this study, we used LPS-stimulated macrophages as the model to examine if stir-baking alters the anti-inflammatory effects of XF. As shown in Fig. 2, LPS treatment enhanced NO production (*p*<0.01) (Fig. 2a) and increased mRNA expression of iNOS (*p*<0.01) (Fig. 2b) in RAW 264.7 macrophages. Both XF and SBXF water extracts significantly reduced the LPS-elicited NO production (*p*<0.01) (Fig. 2a) and mRNA expression of iNOS (*p*<0.01) (Fig. 2b) in a dose-dependent manner. Interestingly, when compared with XF at 300 μg/ml, SBXF at the same concentration was more potent in the inhibition of NO production (*p*<0.01) (Fig. 2a) and in the reduction of mRNA expression of iNOS (*p*<0.05) (Fig. 2b) in the macrophages. NO is known to be synthesized from L-arginine by nitric oxide synthase (NOS) and plays a pivotal role as a proinflammatory mediator in various diseases [16–18]. iNOS is highly expressed in LPS-activated macrophages [19] and plays a role in the development and maintenance of inflammation and pain [20, 21]. Thus, NO production by iNOS may reflect the degree of inflammation and measurements of these two molecules provide options for assessing the effect of drugs in the inflammatory process [22]. These results showed that stir-baking enhanced the anti-inflammatory effects of XF in vitro.

### Stir-baking altered the chemical profile of XF

**Fig. 3:**
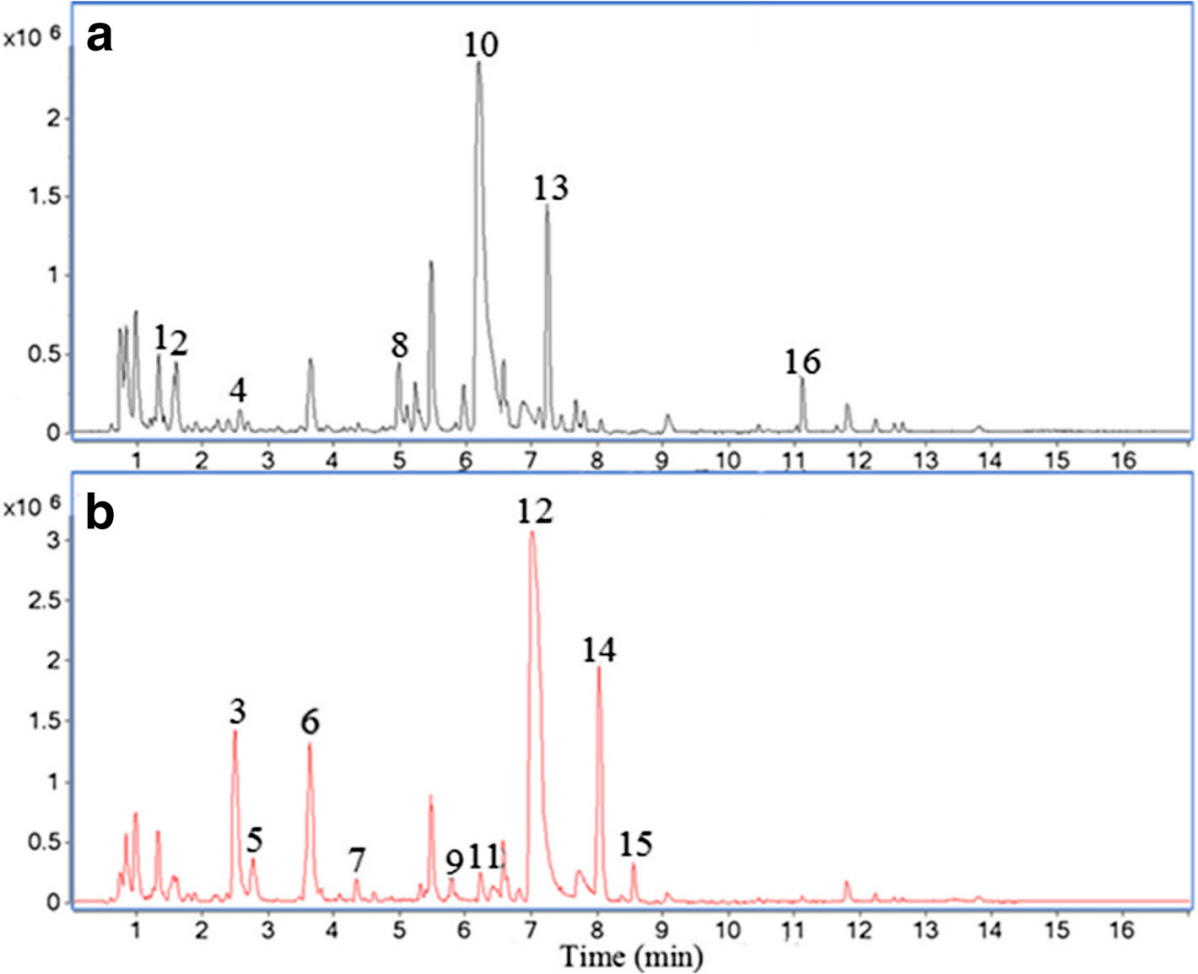
The representative negative base peak intensity (BPI) chromatograms of XF and SBXF. (**a**): Chemical profile of XF detected by UPLC/Q-TOF-MS analyses. (**b**): Chemical profile of SBXF detected by UPLC/Q-TOF-MS analyses

In an attempt to uncover the underlying chemical basis of the reduced cytotoxicity and the enhanced anti-inflammatory effects caused by stir-baking, we compared the chemical profiles of XF and SBXF by UPLC/Q-TOF-MS analyses. As shown in Fig. 3, seven peaks were lower, while nine peaks were higher in SBXF than in XF. Compounds 1, 2, 3, 5 and 7, 9, 11 are two types of isomers, and these compounds were identified by matching with their empirical molecular formulae and mass fragments, as well as the literature data [23 29 ]. Compounds 3, 6, 10, 11, 12 were confirmed as chlorogenic acid, caffeic acid, carboxyatractyloside, 1,5-O-dicaffeoylquinic acid, and atractyloside by the reference standards. Details of the MS of all components, including phenolic acids and water-soluble glycosides, corresponding to individual peaks were shown in Table 1. Seven compounds, 1-O-caffeotannic acid, 3-O-caffeotannic acid, chlorogenic acid, 4-O-caffeotannic acid, 1,3-O-dicaffeotannic acid, 1,4-O-dicaffeotannic acid and 1,5-O-dicaffeotannic acid have been documented to possess anti-inflammatory effects [23].

**Table 1:** Compounds identified from the water extracts of XF and SBXF

Peak no.	t_R_ (min)	Assigned identity	Molecular	Mean measured mass (Da)	Mass accuracy (ppm)	Theoretical exact mass (Da)	Quasi-molecular ion	MS/MS m/z (ESI^−^)	Change trend after processing	References
1	1.368	1-O-caffeoylquinic acid	C_16_H_18_O_9_	353.0866	3.51	353.0873	[M-H]^−^	191	↓	24
2	1.593	3-O-caffeoylquinic acid	C_16_H_18_O_9_	353.0866	8.75	353.0873	[M-H]^−^	191;135	↓*	23
3^a^	2.487	chlorogenic acid	C_16_H_18_O_9_	353.0866	−1.35	353.0873	[M-H]^−^	191	↑**	
4	2.546	UN	C_7_H_12_O_5_	175.0612	−3.78	175.0606	[M-H]^−^		↓	
5	2.763	4-O-caffeoylquinic acid	C_16_H_18_O_9_	353.0866	−6.55	353.0873	[M-H]^−^	173;135	↑*	24
6^a^	3.623	caffeic acid	C_9_H_8_O_4_	179.0350	5.36	179.0344	[M-H]^−^		↑**	27
7	4.349	1,3-O-dicaffeoylquinic acid	C_25_H_24_O_12_	515.1179	3.05	515.1190	[M-H]^−^	353;299,173	↑	25,26
8	4.985	UN	C_25_H_24_O_13_	531.1144	−0.36	531.1131	[M-H]^−^		↓	
9	6.028	1,4-O-dicaffeoylquinic acid	C_25_H_24_O_12_	515.1195	8.85	515.1190	[M-H]^−^	353;335;179	↑	23
10^a^	6.195	carboxyatractyloside	C_31_H_46_O_18_S_2_	769.2110	1.23	769.2047	[M-H]^−^		↓**	12, 28,
11^a^	6.228	1,5-O-dicaffeoylquinic acid	C_25_H_24_O_12_	515.1299	−6.07	515.1190	[M-H]^−^	353;335;191	↑*	25,26
12^a^	7.03	atractyloside	C_30_H_46_O_16_S_2_	725.2155	−0.62	725.2149	[M-H]^−^		↑**	28,29
13	7.239	UN	C_27_H_46_O_20_	689.2536	−3.72	689.2490	[M-H]^−^		↓**	
14	8.032	4'-desulphate-atractyloside	C_30_H_46_O_13_S	645.2594	−1.05	645.2581	[M-H]^−^		↑**	29
15	8.575	UN	C_34_H_44_O_13_	659.2733	−3.57	659.2691	[M-H]^−^		↑*	
16	11.119	UN	C_19_H_30_N_4_	313.2398	0.04	313.2386	[M-H]^−^		↓**	

*P<0.05; **P<0.01; *UN* unidentified

↑increased after processing procedure; ↓decreased after processing procedure

^a^Identified with reference standards

**Fig. 4:**
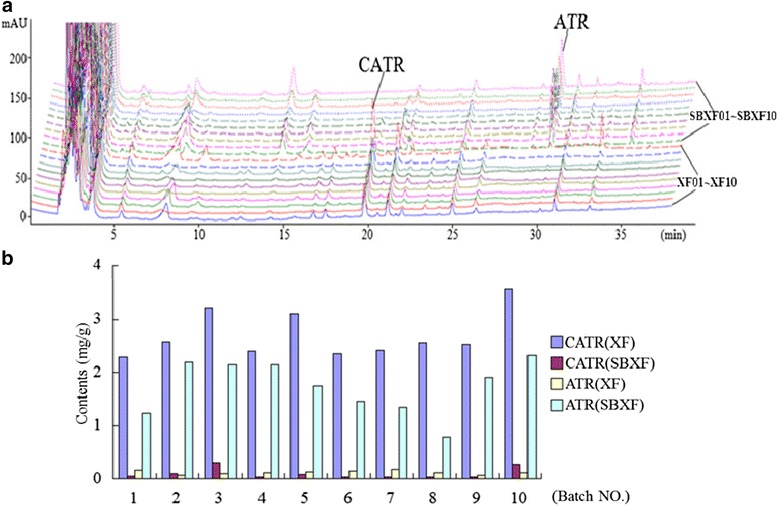
Quantitative analyses of CATR and ATR. (**a**) The typical HPLC chromatograms of ten batches of XF and corresponding SBXF; (**b**) The contents of CATR and ATR in ten batches of XF and corresponding SBXF. CATR: Carboxyatractyloside; ATR: Atractyloside

It has been reported that two water-soluble glycosides CATR and ATR are the toxic components of this herb [11, 12]. In this study, we compared the contents of CATR and ATR in ten batches of XF and corresponding SBXF samples using a HPLC method. Results showed that after stir-baking, the content of CATR was decreased 27.0-fold, however, the content of ATR was increased 13.3-fold in this herb. It was reported that CATR is more toxic than ATR in in vitro and in vivo [30, 31]. Whether CATR in the herb transformed into ATR during stir-baking needs to be further studied. The typical HPLC chromatograms of ten batches of XF and corresponding SBXF were shown in Fig. 4a, and the contents of CATR and ATR in ten batches of XF and SBXF were shown in Fig. 4b.

Chemical and bioactivity studies will be performed to determine whether the changed compounds are responsible for the reduced cytotoxicity and the enhanced anti-inflammatory effects of XF after stir-baking.

## Conclusion

We demonstrated that stir-baking significantly reduced the cytotoxicity and enhanced the anti-inflammatory effects of XF, which support the TCM theory “stir-baking can reduce the toxicity and enhance the efficacy of XF”. We have also found that stir-baking caused alterations of components in XF as determined by an established UPLC/Q-TOF-MS method, which might be responsible for the reduced toxicity and enhanced efficacy afforded by processing.

## Abbreviations

ANOVA: analysis of variance
COX-2: cyclooxygenase-2
DMEM: dulbecco’s modified eagle medium
ESI: electrospray ionization
iNOS: inducible nitric oxide synthase
NO: nitric oxide
TCM: Traditional Chinese medicine
UPLC/Q-TOF-MS: ultra-performance liquid chromatography/quadrupole-time-of-flight mass spectrometry
